# Supplementary material for: HIVprotI: an integrated web based platform for prediction and design of HIV proteins inhibitors
Source: J Cheminform. 2018 Mar 9;10:12. doi: 10.1186/s13321-018-0266-y (PMC5845081; doi:10.1186/s13321-018-0266-y)
Supplement: Supplementary file 2 — Additional file 2. Source code of HIVProtI web server. [file 13321_2018_266_MOESM2_ESM.zip › HIVprotI_Source-code/hivprotI/weka-3-6-6/doc/weka/attributeSelection/CostSensitiveASEvaluation.html]

CostSensitiveASEvaluation


---


|  |  |  |
| --- | --- | --- |
| **Overview** \| **Package** \| **Class** \| **Tree** \| **Deprecated** \| **Index** \| **Help** \| **Weka's home** | |  |
| **PREV CLASS**   **NEXT CLASS** | **FRAMES**    **NO FRAMES**     **All Classes** |  |
| SUMMARY: NESTED \| FIELD \| CONSTR \| METHOD | DETAIL: FIELD \| CONSTR \| METHOD |  |


---


## weka.attributeSelection Class CostSensitiveASEvaluation

```
java.lang.Object
  weka.attributeSelection.ASEvaluation
      weka.attributeSelection.CostSensitiveASEvaluation
```

**All Implemented Interfaces:**: java.io.Serializable, CapabilitiesHandler, OptionHandler, RevisionHandler

**Direct Known Subclasses:**: CostSensitiveAttributeEval, CostSensitiveSubsetEval

---

``` public abstract class CostSensitiveASEvaluation extends ASEvaluation implements OptionHandler, java.io.Serializable ```

Abstract base class for cost-sensitive subset and attribute evaluators.

**Version:**
:   $Revision: 5562 $

**Author:**
:   Mark Hall (mhall{[at]}pentaho{[dot]}com)

**See Also:**: Serialized Form

---

| **Field Summary** | |
| --- | --- |
| `static int` | `MATRIX_ON_DEMAND`             load cost matrix on demand |
| `static int` | `MATRIX_SUPPLIED`             use explicit cost matrix |
| `static Tag[]` | `TAGS_MATRIX_SOURCE`             Specify possible sources of the cost matrix |


**Constructor Summary**

`CostSensitiveASEvaluation()`


| **Method Summary** | |
| --- | --- |
| `void` | `buildEvaluator(Instances data)`             Generates a attribute evaluator. |
| `java.lang.String` | `costMatrixSourceTipText()` |
| `java.lang.String` | `costMatrixTipText()` |
| `java.lang.String` | `defaultEvaluatorString()`             Return the name of the default evaluator. |
| `java.lang.String` | `evaluatorTipText()`             Returns the tip text for this property |
| `Capabilities` | `getCapabilities()`             Returns default capabilities of the classifier. |
| `CostMatrix` | `getCostMatrix()`             Gets the misclassification cost matrix. |
| `SelectedTag` | `getCostMatrixSource()`             Gets the source location method of the cost matrix. |
| `ASEvaluation` | `getEvaluator()`             Get the evaluator used as the base evaluator. |
| `java.io.File` | `getOnDemandDirectory()`             Returns the directory that will be searched for cost files when loading on demand. |
| `java.lang.String[]` | `getOptions()`             Gets the current settings of the subset evaluator. |
| `java.lang.String` | `getRevision()`             Returns the revision string. |
| `int` | `getSeed()`             Gets the seed for the random number generations. |
| `java.lang.String` | `globalInfo()` |
| `java.util.Enumeration` | `listOptions()`             Returns an enumeration describing the available options. |
| `java.lang.String` | `onDemandDirectoryTipText()` |
| `int[]` | `postProcess(int[] attributeSet)`             Provides a chance for a attribute evaluator to do any special post processing of the selected attribute set. |
| `java.lang.String` | `seedTipText()`             Returns the tip text for this property |
| `void` | `setCostMatrix(CostMatrix newCostMatrix)`             Sets the misclassification cost matrix. |
| `void` | `setCostMatrixSource(SelectedTag newMethod)`             Sets the source location of the cost matrix. |
| `void` | `setEvaluator(ASEvaluation newEvaluator)`             Set the base evaluator. |
| `void` | `setOnDemandDirectory(java.io.File newDir)`             Sets the directory that will be searched for cost files when loading on demand. |
| `void` | `setOptions(java.lang.String[] options)`             Parses a given list of options. |
| `void` | `setSeed(int seed)`             Set the seed for random number generation. |
| `java.lang.String` | `toString()`             Output a representation of this evaluator |

**Methods inherited from class weka.attributeSelection.ASEvaluation**

`forName, makeCopies`

**Methods inherited from class java.lang.Object**

`equals, getClass, hashCode, notify, notifyAll, wait, wait, wait`

**Field Detail**

### MATRIX\_ON\_DEMAND

```
public static final int MATRIX_ON_DEMAND
```

:   load cost matrix on demand

    **See Also:**: Constant Field Values

---


### MATRIX\_SUPPLIED

```
public static final int MATRIX_SUPPLIED
```

:   use explicit cost matrix

    **See Also:**: Constant Field Values

---


### TAGS\_MATRIX\_SOURCE

```
public static final Tag[] TAGS_MATRIX_SOURCE
```

:   Specify possible sources of the cost matrix


**Constructor Detail**

### CostSensitiveASEvaluation

```
public CostSensitiveASEvaluation()
```


**Method Detail**

### listOptions

```
public java.util.Enumeration listOptions()
```

:   Returns an enumeration describing the available options.

    :   **Specified by:**: `listOptions` in interface `OptionHandler`
    :   **Returns:**: an enumeration of all the available options.

---


### setOptions

```
public void setOptions(java.lang.String[] options)
                throws java.lang.Exception
```

:   Parses a given list of options.

    Valid options are:

    ```
     -C <cost file name>
      File name of a cost matrix to use. If this is not supplied,
      a cost matrix will be loaded on demand. The name of the
      on-demand file is the relation name of the training data
      plus ".cost", and the path to the on-demand file is
      specified with the -N option.
    ```

    ```
     -N <directory>
      Name of a directory to search for cost files when loading
      costs on demand (default current directory).
    ```

    ```
     -cost-matrix <matrix>
      The cost matrix in Matlab single line format.
    ```

    ```
     -S <integer>
      The seed to use for random number generation.
    ```

    ```
     -W
      Full name of base evaluator.
      (default: weka.attributeSelection.CfsSubsetEval)
    ```

    Options after -- are passed to the designated evaluator.

    :   **Specified by:**: `setOptions` in interface `OptionHandler`
    :   **Parameters:**: `options` - the list of options as an array of strings **Throws:**: `java.lang.Exception` - if an option is not supported

---


### getOptions

```
public java.lang.String[] getOptions()
```

:   Gets the current settings of the subset evaluator.

    :   **Specified by:**: `getOptions` in interface `OptionHandler`
    :   **Returns:**: an array of strings suitable for passing to setOptions

---


### globalInfo

```
public java.lang.String globalInfo()
```

:   **Returns:**: a description of the classifier suitable for displaying in the explorer/experimenter gui

---


### defaultEvaluatorString

```
public java.lang.String defaultEvaluatorString()
```

:   Return the name of the default evaluator.

    :   **Returns:**: the name of the default evaluator

---


### costMatrixSourceTipText

```
public java.lang.String costMatrixSourceTipText()
```

:   **Returns:**: tip text for this property suitable for displaying in the explorer/experimenter gui

---


### getCostMatrixSource

```
public SelectedTag getCostMatrixSource()
```

:   Gets the source location method of the cost matrix. Will be one of
    MATRIX\_ON\_DEMAND or MATRIX\_SUPPLIED.

    :   **Returns:**: the cost matrix source.

---


### setCostMatrixSource

```
public void setCostMatrixSource(SelectedTag newMethod)
```

:   Sets the source location of the cost matrix. Values other than
    MATRIX\_ON\_DEMAND or MATRIX\_SUPPLIED will be ignored.

    :   **Parameters:**: `newMethod` - the cost matrix location method.

---


### onDemandDirectoryTipText

```
public java.lang.String onDemandDirectoryTipText()
```

:   **Returns:**: tip text for this property suitable for displaying in the explorer/experimenter gui

---


### getOnDemandDirectory

```
public java.io.File getOnDemandDirectory()
```

:   Returns the directory that will be searched for cost files when
    loading on demand.

    :   **Returns:**: The cost file search directory.

---


### setOnDemandDirectory

```
public void setOnDemandDirectory(java.io.File newDir)
```

:   Sets the directory that will be searched for cost files when
    loading on demand.

    :   **Parameters:**: `newDir` - The cost file search directory.

---


### costMatrixTipText

```
public java.lang.String costMatrixTipText()
```

:   **Returns:**: tip text for this property suitable for displaying in the explorer/experimenter gui

---


### getCostMatrix

```
public CostMatrix getCostMatrix()
```

:   Gets the misclassification cost matrix.

    :   **Returns:**: the cost matrix

---


### setCostMatrix

```
public void setCostMatrix(CostMatrix newCostMatrix)
```

:   Sets the misclassification cost matrix.

    :   **Parameters:**: `newCostMatrix` - the cost matrix

---


### seedTipText

```
public java.lang.String seedTipText()
```

:   Returns the tip text for this property

    :   **Returns:**: tip text for this property suitable for displaying in the explorer/experimenter gui

---


### setSeed

```
public void setSeed(int seed)
```

:   Set the seed for random number generation.

    :   **Parameters:**: `seed` - the seed

---


### getSeed

```
public int getSeed()
```

:   Gets the seed for the random number generations.

    :   **Returns:**: the seed for the random number generation

---


### evaluatorTipText

```
public java.lang.String evaluatorTipText()
```

:   Returns the tip text for this property

    :   **Returns:**: tip text for this property suitable for displaying in the explorer/experimenter gui

---


### setEvaluator

```
public void setEvaluator(ASEvaluation newEvaluator)
                  throws java.lang.IllegalArgumentException
```

:   Set the base evaluator.

    :   **Parameters:**: `newEvaluator` - the evaluator to use. **Throws:**: `java.lang.IllegalArgumentException` - if the evaluator is of the wrong type

---


### getEvaluator

```
public ASEvaluation getEvaluator()
```

:   Get the evaluator used as the base evaluator.

    :   **Returns:**: the evaluator used as the base evaluator

---


### getCapabilities

```
public Capabilities getCapabilities()
```

:   Returns default capabilities of the classifier.

    :   **Specified by:**: `getCapabilities` in interface `CapabilitiesHandler` **Overrides:**: `getCapabilities` in class `ASEvaluation`
    :   **Returns:**: the capabilities of this classifier **See Also:**: `Capabilities`

---


### buildEvaluator

```
public void buildEvaluator(Instances data)
                    throws java.lang.Exception
```

:   Generates a attribute evaluator. Has to initialize all fields of the
    evaluator that are not being set via options.

    :   **Specified by:**: `buildEvaluator` in class `ASEvaluation`
    :   **Parameters:**: `data` - set of instances serving as training data **Throws:**: `java.lang.Exception` - if the evaluator has not been generated successfully

---


### postProcess

```
public int[] postProcess(int[] attributeSet)
                  throws java.lang.Exception
```

:   Provides a chance for a attribute evaluator to do any special
    post processing of the selected attribute set.

    :   **Overrides:**: `postProcess` in class `ASEvaluation`
    :   **Parameters:**: `attributeSet` - the set of attributes found by the search **Returns:**: a possibly ranked list of postprocessed attributes **Throws:**: `java.lang.Exception` - if postprocessing fails for some reason

---


### toString

```
public java.lang.String toString()
```

:   Output a representation of this evaluator

    :   **Overrides:**: `toString` in class `java.lang.Object`
    :   **Returns:**: a string representation of the classifier

---


### getRevision

```
public java.lang.String getRevision()
```

:   Returns the revision string.

    :   **Specified by:**: `getRevision` in interface `RevisionHandler` **Overrides:**: `getRevision` in class `ASEvaluation`
    :   **Returns:**: the revision


---


|  |  |  |
| --- | --- | --- |
| **Overview** \| **Package** \| **Class** \| **Tree** \| **Deprecated** \| **Index** \| **Help** \| **Weka's home** | |  |
| **PREV CLASS**   **NEXT CLASS** | **FRAMES**    **NO FRAMES**     **All Classes** |  |
| SUMMARY: NESTED \| FIELD \| CONSTR \| METHOD | DETAIL: FIELD \| CONSTR \| METHOD |  |


---
